# Viral information

**DOI:** 10.1007/s10539-012-9344-0

**Published:** 2012-10-31

**Authors:** Forest Rohwer, Katie Barott

**Affiliations:** Department of Biology, San Diego State University, 5500 Campanile Drive, San Diego, CA 92182 USA

**Keywords:** Virus, Phage, Information, Ecology, Evolution

## Abstract

Viruses are major drivers of global biogeochemistry and the etiological agents of many diseases. They are also the winners in the game of life: there are more viruses on the planet than cellular organisms and they encode most of the genetic diversity on the planet. In fact, it is reasonable to view life as a viral incubator. Nevertheless, most ecological and evolutionary theories were developed, and continue to be developed, without considering the virosphere. This means these theories need to be to reinterpreted in light of viral knowledge or we need to develop new theory from the viral point-of-view. Here we briefly introduce our viral planet and then address a major outstanding question in biology: why is most of life viral? A key insight is that during an infection cycle the original virus is completely broken down and only the associated information is passed on to the next generation. This is different for cellular organisms, which must pass on some physical part of themselves from generation to generation. Based on this premise, it is proposed that the thermodynamic consequences of physical information (e.g., Landauer’s principle) are observed in natural viral populations. This link between physical and genetic information is then used to develop the Viral Information Hypothesis, which states that genetic information replicates itself to the detriment of system energy efficiency (i.e., is viral in nature). Finally, we show how viral information can be tested, and illustrate how this novel view can explain existing ecological and evolutionary theories from more fundamental principles.

## Viruses as information

Viruses are the only biological entities that replicate purely as information. When a virus enters its host, the virion completely disassembles and the nucleic acid is copied into new genomes, which are then packaged and released as new virions. Physically, there is nothing in the original form of the virion that has to be passed on from one generation to another. Not one single molecule, atom, or quark must be transferred between the old and new. The only thing that must be moved between viral generations is the information to build the next set of viruses. The rest of biology operates differently. Every new cell physically shares all of its molecules with the original mother cell at the moment of division.

Here we propose the Viral Information Hypothesis, which argues that:

1. Physical information is about position in the Universe.
2. Biology creates physical information by changing the position of matter, effectively working as Maxwell’s Demon.
3. Viral information converts different types of physical information into itself at the cost of overall energetic efficiency.
4. There is a thermodynamic cost to destroying physical information, which is quantified by Landauer’s Principle. Extremely large populations like viruses experience selection at the Landauer limit and this is observable.

## Welcome to the viral world

Humans observe nature at the meso-scale (e.g., mm to km). Our brains are good at processing this sort of data, from observing blossoming cherry trees, to scuba diving on a coral reef, to measuring the beaks of finches. But our senses have led us astray because until recently we have been overlooking most of life. On the cherry tree’s blossom, roots, branches, and leaves are millions of viruses and their microbial prey. When swimming over a coral reef, every milliliter of seawater is home to ten million viruses (Bergh et al. 1989; Hara et al. 1991; Fuhrman 1999) and every surface, including the mucus on corals and fish, is covered by even more viruses (Wilson et al. 2005; Marhaver et al. 2008; Patten et al. 2008; Willner et al. 2010). And much of the DNA flying about in Darwin’s famous finches actually belongs to microbes and viruses.^1^

Viruses are particularly easy to overlook because they are completely outside our sensory range. This is a problem, because by missing the virosphere biologists have effectively ignored the most abundant and diverse biological entities on Earth. Conservatively, there are 1.0 × 10^31^ of them. This is based on estimates of ~1.0 × 10^30^ microbes on the planet (Whitman et al. 1998) and an average of ~10 viruses per prokaryotic cell (Weinbauer 2004). An alien visiting our planet, given a different sensory range that could directly detect viruses, would likely consider them the dominant form of life. (Note to reader: if you are fluent in the history and biology of viruses, feel free to skip the following section as we review these topics.)

How do we know that there are this many viruses? Initially, they were counted using electron microscopes (Bergh et al. 1989). Now they are routinely counted using epifluorescent microscopy (Noble and Fuhrman 1998). For example, to enumerate the viruses in a milliliter of seawater, the sample is pulled through a glass filter with 0.02 micron pores (small enough to capture viruses). Then the filter is treated with a DNA stain that lights up under fluorescent light on the microscope. Technically, what biologists actually count are virus-like particles (VLPs). A VLP is something that looks like a virus but has not formally been characterized and shown to act like a virus; that is to infect and then replicate inside a host cell. Even though viruses are incredibly small, 10^31^ make them a huge crowd. If you line up all the viruses in single-file, the line would reach a thousand times across our home galaxy.

While the total number of viruses is enormous, what is really incredible is their dynamics (Weinbauer 2004). Our best estimates are that every week 10^31^ viruses fall apart and 10^31^ new ones are made to replace them. This means that roughly 1.7 × 10^25^ new viruses are produced every second. For each new virus, approximately 50,000 base pairs of DNA have to be synthesized (Steward et al. 2000). Thus, each second more than 10^30^ base pairs of viral DNA are made on planet Earth. Since the vast majority of these viruses infect microbes (Bacteria and Archaea, two of the three domains of life), the making of these viruses entails the death of approximately 10^24^ microbial cells each second. This enhances the microbial diversity and productivity of ecosystems. It also is a huge factor in global energy and nutrient cycling (Fuhrman 1999). The point of these exercises is to show just how numerous, massive, and dynamic these 10^31^ viruses really are. When considering the virosphere, extremely unlikely events become probabilistic certainties.

Even though viruses dominate our home in the universe, most people consider them only when they cause some sort of disease.^2^ But in fact, most viruses are actually phage: viruses that infect the Bacteria. In 1915 the Englishman Frederick Twort discovered an “ultra-microscopic virus” that converted bacteria into fine granules (Twort 1915). In his usage, the word ‘virus’ seems to have meant simply an infectious agent. He wrote about “a minute bacterium that will only grow on living material…or a form of life more lowly organized than the bacterium” (1915: 1242). The virus was destroyed at 60 °C and could not be cultured except on the bacteria. “On the whole it seems probable, though by no means certain, that the active transparent material is produced by the micrococcus, and since it leads to its own destruction and can be transmitted to fresh healthy cultures, it might almost be considered as an acute infectious disease of micrococci” (1915:1243). That is, the bacteria were getting sick.

The French-Canadian Felix d’Herelle went further and showed that a filterable “antagonistic microbe” capable of killing the bacteria *Shigella dysenteriae* was isolatable from patients who developed enteritis following dysentery infections (d’Herelle 1917). He performed the first plaque assays and showed that titers of this agent were highest during patient recovery. Culturing the agent required living dysentery bacteria, but under these conditions the agent could be cultured through 50 successive transfers. d’Herelle wrote: “the disappearance of the dysentery bacilli is coincident with the appearance of an invisible microbe…This microbe, really a microbe of immunity, is an obligate **bacteriophage**”—the first use of the term (d’Herelle 1917: 159).

Bacteriophage means “bacteria eating” and is usually shortened to *phage*. They are a subclass of viruses that infect the Bacterial domain of life (Woese et al. 1990). The early virus hunters quickly realized the phage were very diverse, with each one finicky about which host it would infect. This specificity of phages for specific strains of bacteria was one of the early ways of microbiological identification, which was carried out through a procedure called phage typing (Williams and Rippon 1952). Basically, this can be done by culturing bacteria in a test tube and then adding different phage. If the tube clears, it means all the bacteria have been killed by the phage. Using this approach, thousands of phage and their host strains have been classified. Determining host range is one of the most a useful approaches for characterizing the virosphere.

**Fig. 1:**
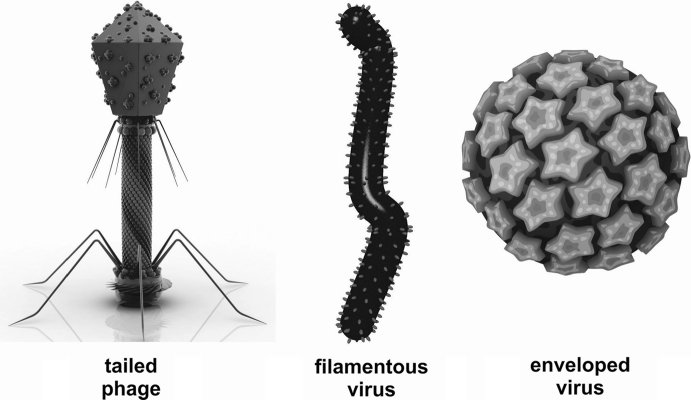
Examples of the main types of viruses: tailed phage that infect bacteria, filamentous viruses that infect all domains of life, and enveloped viruses that infect animal and plant cells. There are actually hundreds of variants on these basic themes and interested readers should look at the International Committee on Viral Taxonomy (ICTV) website and/or Viral Taxonomy books. Of particular interest are the numerous novel virions associated with Archaea viruses

Another way to characterize viruses is to visualize them with an electron microscope (EM) (Bradley 1965). Viruses outside a host cell are called virions and they are some of the most wondrous creatures ever discovered. The archetypical phage looks like a lunar lander, with a protein capsid protecting the double stranded DNA genome (Fig. 1). It has a tail, which is used to transfer the viral genome into the host cell, and tail fibers that help the phage find the correct host (Fig. 1). Viral capsids usually form one of two basic architectures: rods or icosahedra, the size of which can vary by more than an order of magnitude, and which can package genomes that also differ more than 30-fold in size (Fauquet et al. 2005) (Fig. 1). In forming rod-shaped particles, the capsid proteins are arrayed in a helix around the viral DNA or RNA. TMV (tobacco mosaic virus) is the classic example of this shape (Klug 1999). The other common capsid structure is an icosahedron surrounding a nucleic acid core (Fauquet et al. 2005). Among the five thousand-plus phages that had been described and viewed under the EM by 2000, 96 % are “tailed phages” (Ackermann 2001), composed of an icosahedral head containing the genome, and possessing a tail that functions to identify the host and deliver the genome to the cell interior. The tail structures divided the lunar-lander phage into three main groups: the lambda-like phage, which have long, flexible tails; the T7-like phage, which have short, contractile tails; and the T4-like phage, which have long contractile tails (Ackermann 2007). Often the capsid proteins and genomic nucleic acids self-assemble in vitro to form infectious virions (Hung et al. 1969; Kushner 1969; Lebeurier et al. 1977; Klug 1999). Neither additional information nor an energy source is required. Viruses that infect animals and plant often have the icosahedral structure enclosed in an envelope of lipids (Fig. 1).

The virions are exquisitely designed predators that seek out and kill their hosts. Overall, the virions have a slightly negative charge so that they repel each other when the host cell is lysed (Todd 1927; Krueger et al. 1929; Clifton and Madison 1931). This allows them to spread out and avoid entanglement in the lysed cell’s released contents. More subtly, it appears that the charges are arranged so that the virions are actually dipoles (De Groot et al. 1977); that is they have a negative charge around the capsid head and a slightly positive charge at the tails (Serwer and Pichler 1978; Kosturko et al. 1979). This presumably orientates them tail-first when making an attack on the bacterial host cell (which is slightly negative).

During the attack phase, the virion is first electrostatically attracted to the cell’s surface (Krueger 1931; Delbrück 1940). It rolls along the outside and the tail searches for specific receptors. If the host is the correct bacterial species, then the phage will find the receptor and clamp down on it (Heller 1992). When this happens the phage’s tail will drill through host cell’s membranes and cell wall so that the viral genome can be delivered into the cytoplasm (Letellier et al. 2004). To achieve this, the outside of the contractile tails rearrange their molecular structure so that the tube inside the sheath can pierce the cell (Kanamaru et al. 2002; Leiman et al. 2004). This allows for the DNA to be injected with incredible force (Kindt et al. 2001; Letellier et al. 2004). The process is not dissimilar to the secondary jaws of Ridley Scott’s Aliens; terrifying if you happen to be the size of a microbe.

In addition to tails, the phage capsids are often decorated with secondary structures that facilitate the attack. This includes hooks that grab hold of bacterial flagella so that the phage is pulled down to the host (Schade et al. 1967; Lotz et al. 1977). Other molecular accessories probably help the virion survive different environmental conditions or act as camouflage to throw off protective ectoenzymes produced by the host. The constant war between the virion’s capabilities for finding and infecting the cell, and the retaliation by the host, leads to evolutionary dynamics known as Red Queen (Van Valen 1974) and ecological cycles called Lotka-Volterra/Kill-the-Winner (Bratbak et al. 1990).

There were several problems that had to be circumvented in order to study the diversity and dynamics of the global virome. To culture a virus you need to grow its host and at the present time we only routinely cultivate roughly 1 % of the microbes from the environment (Fuhrman and Campbell 1998). And once conditions to culture the microbe are identified, they have to be modified to encourage infection by a virus. Because of these challenges, the culturing route would be a daunting and defeating path to take. What about sequencing the viral DNA? Sequencing of the 16S ribosomal RNA gene (rDNA) is a common technique used to analyze the diversity of microbial communities, and it capitalizes on the high conservation of this one gene amongst all microbes, thereby avoiding comparison of entire genomes to get at community diversity (Pace et al. 1986; Woese 1987). However, it not possible to take a similar approach with viruses because there is no gene in common between all groups (Rohwer and Edwards 2002). To get around this limitation, a technique for shotgun sequencing random fragments from the pool of all of the viral genomes in the community was developed (Breitbart et al. 2002). This approach is called metagenomics.

Analysis of the entire genetic pool (the metagenome) of a sample was first performed on viral communities isolated from seawater in San Diego (Breitbart et al. 2002). This early study showed that the vast majority of viral sequences (80 % or more) were not recognizable using common bioinformatic searches. That is to say that the uncultured viral DNAs were so dissimilar from every single known sequence accumulated in various databases of known viral, bacterial, and eukaryotic sequences (e.g., GenBank) that we have no idea what they do or to whom they belong. Despite the incredible volume of sequences added to the databases since these metagenomes were first sequenced, most viruses remain unknown. On the other hand, microbial metagenomes, which followed closely behind the first viral metagenomes, were much less mysterious with <20 % of sequences not matching anything in the databases (Dinsdale et al. 2008). Because viruses are incredibly abundant, much more so than microbes, and because the majority of the information contained in viral genomes is unknown, viruses are the final frontier of unexplored genomic diversity and are the largest genetic repository that exists. We are left with the question: *why are there so many viruses*?

## Demons and information

Up until this point, we have argued that viruses are extremely abundant, incredibly diverse, and travel through time and space as information. We propose that this relationship between viruses and information is the key to their success, but what does “information” mean? In the communication sense, information is a measure of “surprisal” (Tribus 1961). The greater the surprise at observing an object, the more information it contains. Gold contains more information than does hydrogen (i.e., it is more surprising to find gold). Considering this concept more deeply, it becomes clear that information is actually an accounting of position in the universe. That is, the gold is created by compressing protons, neutrons, and electrons together in space and time. As these particles become locked together, degrees of freedom are lost and a highly unlikely, and therefore a highly informative event, is created. *This organizing of matter is time and space is physical information.*

Physical information does not come for free. The thermodynamic consequence of physical information was first mathematically defined by Rolf Landauer, who calculated that the minimum energy (E) stored in one bit of information was equal to *kT*ln(2), where *k* = Boltzmann’s constant and *T* = temperature in Kelvin (Landauer 1996). Heat released by the erasure of physical information can best be envisioned by invoking Maxwell’s Demon. Originally presented as a challenge to the Second Law of Thermodynamics, the Demon is a hypothetical creature that can pick the “hot” molecules from one container and mover them to another. This creates a temperature differential, which could be used to drive some sort of engine. So, with the right Demon, we can create a perpetual motion machine. It was Leo Szilard who showed that the reason this does not happen is because the Demon is actually gaining information about the relative position of the molecules (Szilard 1929). This realization killed the perpetual motion machine and Maxwell’s challenge to the Second Law of Thermodynamics.

**Fig. 2:**
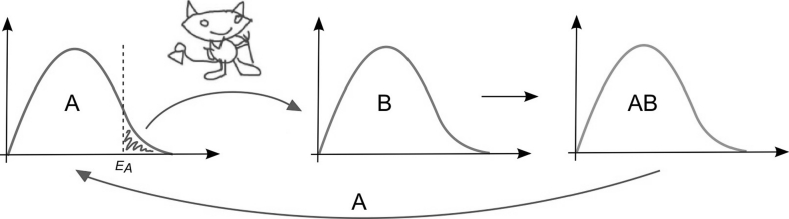
Illustration of Maxwell’s Demon and Landauer’s principle. The Demon/enzyme selectively picks “A” molecules with sufficient energy to react with reactant “B”, which leads to product “AB”. This process slightly cools the “A” population. This loss of heat is put back into the system by the surrounding Universe. During degradation/erasure of “AB”, “A” goes back into its population and this heat can be measured using methods like isothermal calorimetry

Now consider a Maxwell’s Demon in a biochemical system (Fig. 2). At a certain temperature, the reactant molecules “A” have different velocities, as described by Boltzmann’s distribution. The fastest/hottest “A” molecules are on the right side of the distribution. For our purposes, the molecules above the activation energy (E_A_) are the ones with sufficient velocity to be active in a chemical reaction. Now imagine a Maxwell’s Demon that selectively picks “A” molecules within the E_A_ population and passes them to a second reactant pool “B”. This creates the product and effectively traps both molecules in product “AB”. In doing so, the demon has increased the information of the system. When “AB” degrades into its components, “A” will re-enter the original population and heat it up.^3^ This increase in temperature is described by Landauer’s Principle (Landauer 1996; Toyabe et al. 2010).

We propose that biology behaves as Maxwell’s Demon, where the Demons are enzymes that selectively grab E_A_ molecules to form products. This creates physical information, which can be used to do work (Toyabe et al. 2010; Bérut et al. 2012). The one caveat to this *work*-*from*-*information* schema is that it requires elaborate scaffolds like a computer. We suggest that genetic information is the set of instructions to construct the scaffold for Maxwell’s Demons such that they convert different types of physical information into more instances of itself. This new information has a thermodynamic cost when it is erased and the amount of heat released by the destruction of information is also described by Landauer’s Principle (Landauer 1996; Toyabe et al. 2010). It should be possible to observe the link between physical information and thermodynamics and use it to better understand biology and in particular the success of viruses.

## Viral versus physical information

**Fig. 3:**
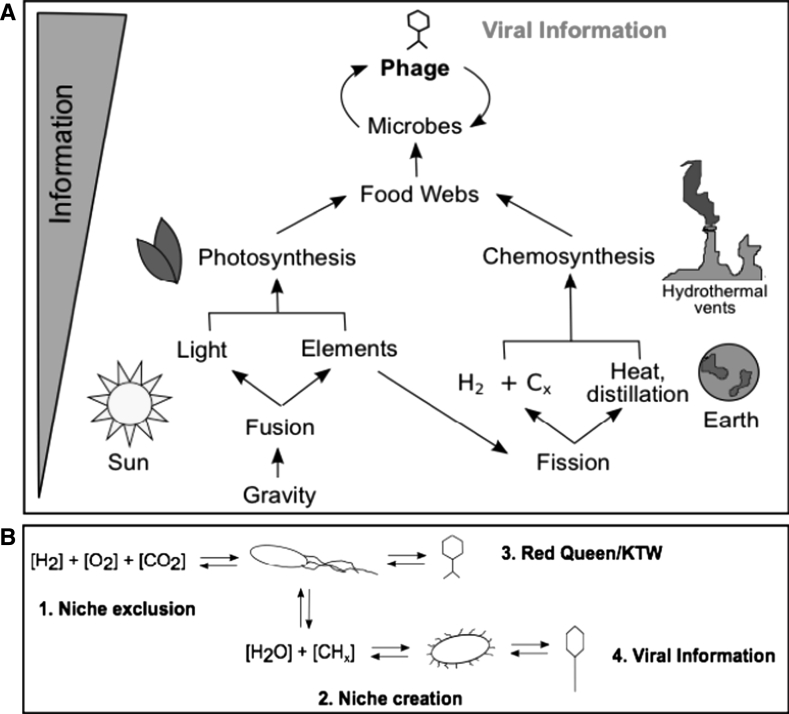
From gravity to viral information: dust to phage. **a** Schematic of how gravity leads to viral information. **b** Schematic of how viruses shape ecology (1-3) and evolution (3), leading to diversification and an increase of viral information (4)

Let us compare and contrast physical and viral information. Gravity organizes the physical properties of the universe. Gravity clumps matter, which enhances the importance of the other three fundamental interactions. By organizing matter in time and space, gravity creates physical information. The cloud of subatomic particles from the Big Bang could have spread out evenly throughout the universe. Instead, small imperfections allowed gravity to pull some particles together; and these attracted others. Accretion discs developed and collapsed into stars, where gravity fused the matter together forming heavier elements and led to the production of electromagnetic radiation. These processes increase the physical information content within the universe through strictly physical processes (Fig. 3a). Gravity also reinforces itself—bigger things attract more objects, creating a positive feedback loop. Biology also reinforces itself by organizing compounds and concentrating them. Just like gravity, life creates organization of particles in the universe through juxtaposition and rearrangement. The organization of matter by biology leads to viral information because it converts physical information into itself at the cost of maximal efficiency from a thermodynamic point of view.

## Viral information and the rest of biology

The two primary sources of physical information used for conversion into viral information are electromagnetic radiation from fusion (the basis for phototrophy) and the redox byproducts of fission (the basis for chemotrophy) (Fig. 3a). Let us examine the largest biome on earth (potentially), the deep, hot biosphere that exists within the Earth’s crust (Gold 1992; Chapelle et al. 2002), as an example of how the viral information feedback might work. In this ecosystem, a very simple energy source, split water, provides the energy to create cellular biomass. At the temperature and pressure of this system, the only known predators of the microbial inhabitants are the viruses. From this simple food web, the main “rules” of ecology and evolution are apparent (Fig. 3b). These are (1) niche exclusion, (2) niche creation, and (3) Red Queen/Kill-the-Winner (KTW) dynamics, which ultimately result in and are driven by (4) viral information.

In the first step, one microbial population makes a living by using up resources from the local environment (e.g., split water). This leaves the system depleted of these items, generating competition and niche exclusion; the microbe that exploits these resources the fastest wins. At the same time, the viruses in the system essentially punish the most successful microbe by killing it (Bratbak et al. 1990; Rodriguez-Brito et al. 2010). Viral lysis releases cellular debris into the surrounding environment, and the new microbes that capitalize on this new set of resources then begin the process anew, excluding others from their new niche, creating a new set of waste products and resources, and feeding a new population of viruses. Effectively, the viruses are creating conditions to replicate themselves.

The pressure of predation also leads the microbe to alter parts of itself to avoid viral recognition (i.e., Red Queen Dynamics, or running to stay in the same place), while the virus adapts to recognize the new microbes. In other words, viruses drive the evolution of microbial genomes and niches, ultimately leading to the increase of viral information. Since viruses can sample more sequence space, they wind up generating the greatest amount of new genetic information, which can then be passed along to the host microbes through horizontal gene transfer (HGT). Ultimately, the community is converting physical information into genetic information. We hypothesize that this step is viral because it is done at a great thermodynamic inefficiency; that is a lot of waste heat is produced. Using the rule of thumb that each trophic transfer loses 90 % of the heat, each joule of viral information gained costs the system 100 J of physical information.

## Measuring viral information

**Fig. 4:**
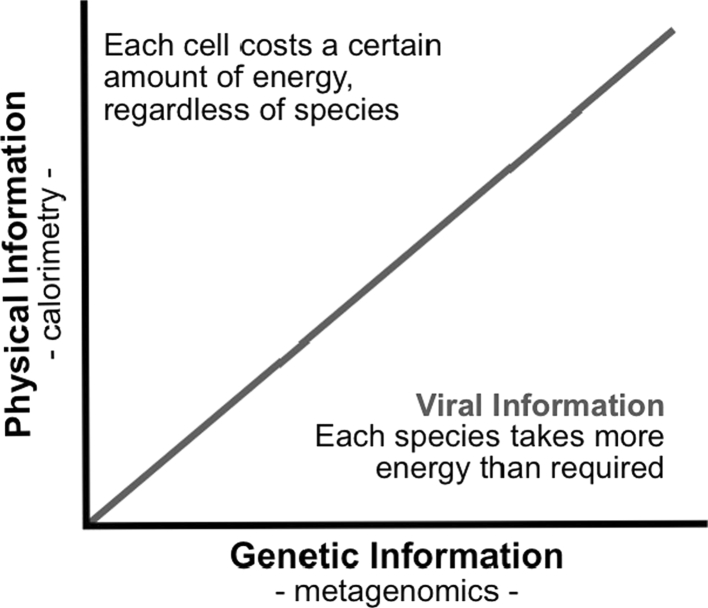
Searching for viral information. The *line* indicates where the amount of physical information and genetic information contained within cells are equal. Communities above the line contain more physical than genetic information due to low genetic diversity (few species but many individuals), with each individual requiring a certain amount of energy regardless of its genetic composition. Communities below the line contain more genetic information. It is here that the energetic cost of information becomes apparent, and where we expect to find viral information

The destruction of physical information, as discussed above, results in the release of heat according to Landauer’s Principle. This heat can be measured by calorimetry. Specifically, isothermal calorimetry tells us about the conversion rate of physical information of a community into heat.^4^ Genetic information of the same community can also be measured, in this case by sequencing the DNA. Based on these two techniques, we propose the following experiment where physical information is followed using calorimetry, and genetic information is followed using metagenomics. When the two are plotted as shown in Fig. 4, we propose that a community dominated by viral information occurs in the lower right region of the graph where genetic information is made at the expense of thermodynamic efficiency (i.e., low conversion to physical information).

Is there any evidence that viral information is real? Djamali and colleagues used isothermal calorimetry to study the heat released by marine microbial and viral communities (Djamali et al. 2012). In this experiment, viruses lowered the standing stock of the cellular component by ~25 %. At the same time, viruses increased the work output of the system by over 200 %. The decline in cell numbers coupled with the increase in diversity looks very much like viral information. Future experiments of this type offer a framework for testing the Viral Information Hypothesis.

## Observations of viral information in nature

As one possible example of the consequences of viral information in nature, let us consider the global conservation of viral sequences. Specific PCR “hunts” for the same virus and/or virally encoded genes have shown that those viruses/viral genes are relatively common all over the world (Breitbart et al. 2004; Short and Suttle 2005; Casas et al. 2006). For example, PCR primers were designed to specifically to amplify two viral sequences named HECTOR and PARIS (Breitbart et al. 2004). These so-called PUP sequences (Polymerases from Uncultured Podophage) were present in most environments investigated and were found to be essentially identical (>99 % conserved at the nucleotide level). Similarly, metagenomic samples have found exactly identical, overlapping viral sequences from widely dispersed parts of the ocean (Angly et al. 2006). Finally, genomic sequencing of phage has identified exactly matching sequences in very different phage genomes (Graham Hatfull, personal communication).

**Fig. 5:**
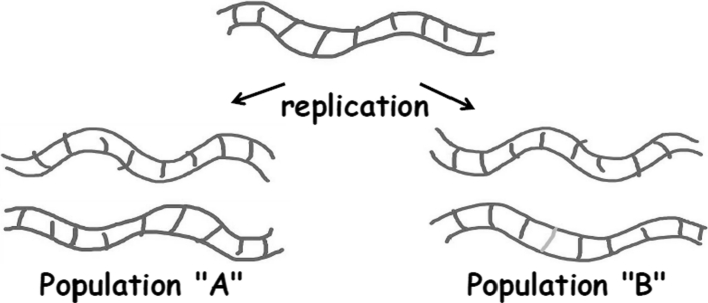
The Landauer limit and mutations. A mutation in a DNA population creates at least 2 bits of Physical Information. It costs an extra 3–6 × 10^−21^ J to erase the “B” population

The widespread occurrence of nearly identical sequences across the planet requires an explanation. We hypothesize that this extremely faithful global conservation is due to the energy cost associated with information erasure. As we have seen, there are literally an astronomical number of viruses on the planet. It is estimated that each viral population (that is the number of individuals of the same species) is 10^23^. If each virus in a population has a difference of one bit of information, then the heat released by destroying that additional information would be 1,800 J via Landauer’s Principle (Fig. 5). In other words, a viral population that has one mutation per genome replication costs 1.8 kJ more to replicate than a viral population that has no mutations. Over the course of a year, the amount of energy required by the mutating viral population versus the non-mutating population is approximately 100 kJ, assuming that the whole population is replaced once a week. Over a billion years, this is 10^14^ J, which is roughly equivalent to the amount of energy released by an atomic bomb.^5^ Energetically efficient populations outcompete those that are less efficient (Meysman and Bruers 2007; Vallino 2010); therefore, populations of viruses with reduced mutations rates will outcompete those with higher mutation rates, all other things being equal.

The extra energetic costs of physical information associated with a mutation might explain why identical viral sequences are observed on a global scale. Physical information in the sense of a mutation is an extremely small selection pressure and *we hypothesize that the Landauer limit is the smallest force of selection*. Because of their “information only” life styles, it is easier to observe the thermodynamic consequences of information in viral communities.^6^ Furthermore, we only observe this in Nature because it is extremely hard to raise 10^23^ phage (or any other biological entity) in the laboratory.

## Conclusion

Envisioning the biosphere as a massive system that ultimately feeds viruses helps us address a major outstanding question: why is biological diversity dominated by viruses? This question would not have even occurred to earlier biologists, simply because they did not know the extent of the virosphere. Modern biology, however, needs to incorporate this natural phenomenon into its canon. The Viral Information Hypothesis has the potential to synthesize ecology and evolutionary theory by incorporating the viruses with the rest of biology in a thermodynamic framework.

## Footnotes

1 Doing some back-of-the-envelope calculations for the zebra finch, *Taeniopygia guttata*, we estimated that about 10 % of the total DNA in a finch is microbial or viral.

2 Attempts to identify the bacteriological pathogen of diseased tobacco plants led to the identification of the first virus by Beijerinck in 1898. The infectious agent was described as a filterable *contagium vivum fluidum,* and was later named the tobacco mosaic virus (Bos 1999).

3 Sometimes it is easier to think of this as only two molecules of A. When one molecule is taken away, the system gets colder. And when the A molecule returns to the system, it gets hotter.

4 In this sense, physical information is the 1/S, where S is entropy. We prefer to use "information" for our accounting of position, because it better explains the concept. However it is completely compatible to express this in terms of S.

5 Remember this competition is actually occurring locally for small parts of the total population.

6 The viruses may not be the only place where Landauer’s limit can be observed. Ultraconserved Elements (UCE) occur in plant and animal genomes. There are 481 UCEs (200 bp) between human, mouse and rats, which represents 300 million years of evolution. Reneker et al. identified large numbers of UCEs (>100 bp) shared between plants and animals (Reneker et al. 2012).
